# Effect of Undernutrition and Obesity on Clinical Outcomes in Adults with Community-Acquired Pneumonia

**DOI:** 10.3390/nu14153235

**Published:** 2022-08-07

**Authors:** Diego Viasus, Valentina Pérez-Vergara, Jordi Carratalà

**Affiliations:** 1 Department of Medicine, Division of Health Sciences, Universidad del Norte and Hospital Universidad del Norte, Barranquilla 081001, Colombia; 2 Department of Infectious Diseases, Bellvitge University Hospital, Bellvitge Biomedical Research Institute (IDIBELL), University of Barcelona, 08907 Barcelona, Spain; 3 Centro de Investigación Biomédica en Red de Enfermedades Infecciosas (CIBERINFEC), Instituto de Salud Carlos III, 28029 Madrid, Spain

**Keywords:** community-acquired pneumonia, influenza, malnutrition, mortality, obesity, undernutrition

## Abstract

Malnutrition comprises two groups of conditions: undernutrition and overweight or obesity. It has been associated with a high risk of contracting infectious diseases and with elevated mortality rates. Community-acquired pneumonia (CAP) is one of the most common infectious diseases worldwide and its prognosis is affected by a large number of recognizable risk factors. This narrative review updates the information on the impact of malnutrition, including both undernutrition and obesity, on the risk and prognosis of adults with CAP. Studies of CAP that have evaluated undernutrition have applied a variety of definitions when assessing the nutritional status of patients. Undernutrition has been associated with unfavorable clinical outcomes, such as prolonged hospital stay, need for intensive care unit admission, and mortality; in contrast, most published studies have found that increased body mass index is significantly associated with higher survival in patients with CAP. However, some authors have presented divergent results, mainly in relation to the etiology of CAP (bacterial versus viral). Influenza infection, caused by influenza A (H1N1) pdm09, has been associated with worse prognosis in obese patients. The current data underscore the need for larger studies to examine the physiological mechanisms that explain the differential impact of malnutrition on outcomes. Achieving a better understanding may help to guide the design of new interventions to improve prognosis.

## 1. Introduction

According to the World Health Organization (WHO), the term malnutrition encompasses two groups of conditions: undernutrition (wasting, stunting, and micronutrient deficiencies) and overweight, obesity, and diet-related noncommunicable diseases [1]. In 2014, it was estimated that approximately 462 million adults suffered from undernutrition, while 1.9 billion were overweight or obese. Today, one in three people worldwide are thought to suffer from one or another form of malnutrition [2]. The impact of malnutrition is serious and lasting, and affects the economic, social, and medical wellbeing of both individuals and families all over the world [1,2].

Malnutrition is associated with a higher risk of illnesses, such as cardiovascular diseases, some cancers, and infections, including community-acquired pneumonia (CAP) [2,3,4]. Infections also contribute to malnutrition, creating a vicious circle. Nutritional status of a sick person is aggravated by malabsorption, loss of appetite, and diversion of nutrients for the immune response [4]. Malnutrition is also related with more severe courses of infection and higher mortality rates [3,4]. CAP is one of the most common infectious diseases worldwide and is associated with high morbidity and mortality [5,6]. CAP is defined as an acute infection of the pulmonary parenchyma not acquired in a hospital or a long-term care facility, characterized by signs and symptoms such as fever, cough, pleuritic pain, respiratory distress, and new infiltrates in diagnostic images [7,8]. Importantly, studies have shown that the burden of CAP assessed by hospital admissions has increased over the years [9,10,11]. De Miguel-Díez et al. [10] performed a retrospective study using the National Hospital Discharge Database to examine trends in incidence and outcomes of CAP in Spain from 2004 to 2013. The incidence of CAP hospitalizations increased from 142.4 cases per 100,000 inhabitants in 2004 to 163.87 in 2013, even when the analysis was performed in both sexes. Moreover, overall mortality rates of 5% to 18% have been described among hospitalized patients with CAP, and up to 30% to 50% in patients requiring intensive care unit (ICU) admission [6].

Undernutrition and obesity have frequently been reported in patients with CAP and have been associated with variable short- and long-term outcomes, such as clinical failure, and 30-day or 1-year mortality [12,13,14]. The prognosis of patients with malnutrition and CAP appears to differ according to the patient population, the setting, and the outcome measure assessed. Studies have documented that undernutrition has been related with worse prognosis in patients with CAP, while obesity has been associated with a lower risk of mortality [12,13,14,15,16]. However, the results of the studies vary depending on the etiology of CAP [17].

## 2. Methods

A considerable number of recognizable risk factors may impact prognosis in CAP patients [18]. Although recent studies have found that nutritional assessment at the time of hospital admission and appropriate treatment are important aspects of patient care in medical wards [19], most studies evaluating risk factors for complications in CAP and severity scales do not include patients’ nutritional status [8]. This narrative review updates the information regarding the impact of malnutrition, including undernutrition and obesity, on prognosis in adults with an episode of CAP. We searched PubMed/MEDLINE from inception to May 2022. Our search also included other relevant publications from references of included articles. Search terms that we used include “community-acquired pneumonia”, “malnutrition”, “undernutrition”, “obesity”, “overweight”, and “influenza”.

There are several indicators that are used to measure nutritional status, such as serum biochemical levels, functional tests (e.g., handgrip strength), body composition (e.g., bioelectrical impedance analysis, dual-energy X-ray absorptiometry, or isotope dilution methods), or dietary intake (e.g., food recall, food frequency questionnaires, or monitor food intake). Moreover, the commonly used anthropometric measurements are weight and height. Other body composition measurements that are used include various body circumferences and skinfold thicknesses (biceps, triceps, subscapularis) [20,21].

Undernutrition is defined as an insufficient intake of energy and nutrients. Terms such as protein-energy malnutrition, micronutrient deficiencies, and other names such as kwashiorkor and marasmus have been used [1,20]. Recently, diagnostic criteria for undernutrition have been defined by European Society of Clinical Nutrition and Metabolism (ESPEN) [21]. First, subjects at risk of undernutrition should be identified by validated screening tools. Second, body mass index (BMI) < 18.5 kg/m^2^ or the combined finding of unintentional weight loss (mandatory) and at least one of either reduced BMI or a low fat-free mass index should be used to define malnutrition. On the other hand, obesity is defined as an abnormal or excessive accumulation of fat that presents a health risk. Obesity has been considered as a BMI greater than 30 kg/m^2^ [1,21]. However, some authors have described the limitations of BMI to define nutritional status since it does not reflect adiposity and body composition (and its distribution), suggesting that the definition of obesity should consider excess body fat as the main characteristic of this disease [22].

## 3. Malnutrition and the Risk for Developing CAP

### 3.1. Undernutrition and Risk of CAP

Almirall et al. [23] performed a systematic review of observational studies to establish the risk factors for CAP. Undernutrition was a strong predictor of CAP. Importantly, definition of undernutrition in studies encompassed hypoalbuminemia, hypoproteinemia, malnourishment, or a low nutritional score. In this regard, a retrospective case-control study of adults ≥ 65 years was conducted to investigate whether pharmacotherapy was associated with the increased risk for CAP after the adjustment of confounding factors [24]. Lower serum albumin levels increased the risk of CAP by decreasing a gram per deciliter (OR: 2.89, 95% CI: 2.01–4.16). Similarly, Riquelme et al. [25] also conducted a case-control study in the elderly (over the age of 65 years) to establish risk factors for CAP. The investigators found that large-volume aspiration and a poor nutritional status (low serum albumin) were the independent risk factors for developing CAP. Interestingly, in the univariate analysis, low serum albumin, malnourishment, low serum prealbumin, and hypoproteinemia were related with higher risk for CAP. Finally, another study evaluated environmental factors of CAP hospitalization in older adults [26]. Poor nutritional score was associated with a greater risk of pneumonia hospitalization in multivariable analysis (OR: 1.83, 95% CI: 1.19–2.80). Nutritional assessment was conducted using a 15-item adaptation of the Nutrition Screening Initiative and the Mini Nutritional Assessment.

### 3.2. Obesity and Risk of CAP

Because obesity produces changes in the immune response and lung function, studies have evaluated it as a risk factor for developing CAP. A meta-analysis did not show a statistically significant association between the risk of developing CAP and overweight and obesity (RR 0.89, 95% CI 0.8–1.03 and RR 1.03, 95% CI 0.8–1.3 respectively) [27]. However, the results of the studies are controversial. Some have shown that excessive weight gain increases the risk of CAP and aspiration pneumonia [28,29,30]. A prospective study showed that men who gained more than 20 kg had twice the risk of developing CAP than those who maintained their weight. In women, a direct association was found between BMI > 25 kg/m^2^ and the risk of developing CAP [29]. On the other hand, in a case-control study of risk factors for CAP in German adults, Schnoor et al. [31] observed that overweight people have a reduced risk of developing the disease (OR: 0.6, 95% CI: 0.5–0.7).

### 3.3. Obesity and Risk of Influenza

A 13-year retrospective study including 104,665 adults in Ontario, Canada, was conducted to determine the association between obesity and outpatient visits for acute respiratory infections during influenza seasons (1996–1997 to 2008–2009). Increased BMI was associated with higher rates of outpatient visits during both influenza and non-influenza periods. Severely obese individuals presented the highest rates of outpatient visits (RR 1.19; 95% CI: 1.12–1.25). The researchers suggest that the effect of obesity is not specific to influenza infection, and that increased BMI may raise susceptibility to other viral and bacterial respiratory pathogens that cause acute respiratory infections [32]. A similar study evaluated the association between obesity and respiratory hospitalizations during seasonal influenza epidemics [33], finding that patients with obesity had a higher risk of hospitalization for respiratory causes during influenza seasons, and that in the severely obese the risk was higher even in those without comorbidities. Moreover, Neidich et al. [34] found that vaccinated obese adults were twice as likely to be infected with influenza or influenza-like illness compared to vaccinated healthy-weight adults. Thus, obesity may be as important a predictor of acute respiratory infections, including influenza, during influenza seasons as other underlying diseases.

Conversely, Coleman et al. [35] conducted a prospective study in a cohort of people who developed acute respiratory illness and required medical care during two influenza seasons and the 2009 pandemic. After adjusting for confounders, neither obesity nor severe obesity was associated with laboratory-confirmed influenza for each season or for all years combined (OR: 0.95, 95% CI: 0.75–1.20 and OR: 1.10, 95% CI: 0.80–1.52, respectively, for obesity and extreme obesity). Similarly, a retrospective cohort study was conducted in the UK to assess the possible association of BMI with the incidence of influenza-associated pneumonia [36]. This study included a total of 1,074,315 patients, of whom 2.2% were underweight and 73.2% were in the BMI reference range or overweight. Underweight patients had higher rates of influenza-like pneumonia and post-influenza pneumonia, but this was not the case for obese patients.

These conflicting findings for obesity and the risk of influenza emphasize the potential differences in risk based on patient populations, settings, and outcome measures studied. However, a recent meta-analysis including a total of seven studies showed that patients with obesity had a significantly higher risk of influenza infection (OR: 1.29, 95% CI: 1.11–1.49) [37].

## 4. Effect of Malnutrition on Clinical Outcomes in CAP

### 4.1. Undernutrition and Prognosis in CAP

Studies that have evaluated undernutrition and prognosis in CAP in adults use different definitions of nutritional status (Table 1) [12,13,38,39,40,41,42,43]. Undernutrition or poor nutritional status have been associated with unfavorable clinical outcomes in adult patients with CAP [12,13,38,39,40,41,42,43], such as prolonged length of hospital stay (LOS) [12,38,39,41], need for ICU admission [42], or mortality [12,13,39,40,42,43]. In a retrospective cohort study in patients with CAP in Japan carried out between 2014 and 2018, Shimizu et al. [41] associated severe undernutrition in patients ≥ 70 years with higher 30-day in-hospital mortality and 30-day readmission, and prolonged LOS. However, in patients < 70 years, undernutrition was only associated with prolonged LOS. Similarly, another study in CAP patients concluded that low BMI and arm muscle area were associated with increased mortality risk in men, and hypoalbuminemia was associated with increased risk of mortality only in women [43]. Recently, underweight or low BMI values were significantly associated with a higher risk of 30-day, 6-month, and 1-year mortality in hospitalized patients with CAP [14]. Interestingly, a retrospective cohort study conducted in the US in patients hospitalized with CAP of viral etiology (influenza, adenovirus, parainfluenza, respiratory syncytial virus, and human metapneumovirus) found that only 5% of the study population was diagnosed with protein-energy malnutrition (PEM), though PEM was associated with increased mortality, septic shock, pulmonary embolism, mechanical ventilation, and prolonged LOS [13]. Another study reported prolongation of fever in patients with CAP when serum albumin levels were low [39].

### 4.2. Obesity and Prognosis in CAP

Although studies of prognosis in CAP have consistently used BMI to define obesity, the cut-off points to classify patients have varied (Table 2) [14,15,16,29,31,44,45,46,47,48,49,50,51,52,53]. Most published studies have found increased BMI to be significantly associated with increased survival in patients with CAP [14,15,16,44,47,48,49,50,52]. A cohort study documented that obesity was associated with decreased 30-day mortality in 7449 hospitalized patients with CAP. The lowest-mortality risk plateaued at a BMI of 35 kg/m^2^. Another important finding of this study was that the mortality benefits of increased BMI were also seen at 6 months and 1 year [14]. Singanayagam et al. [16] found that obesity (BMI ≥ 30 kg/m^2^) was independently associated with decreased 30-day mortality from CAP, a finding that was not explained by differences in disease severity at hospital admission or the need for mechanical ventilation or inotropic support between obese and non-obese patients. Comparable results were reported by Kahlon et al. [48] in a prospective study conducted in Canada. Among 907 patients with CAP, obese patients had significantly lower in-hospital mortality after multivariable logistic regression analysis compared with normal-weight patients; however, neither being underweight nor being overweight was associated with mortality. Finally, in a recent study [49], obesity was also associated with lower all-cause mortality in 763 patients with CAP. Importantly, differences in inflammatory response did not explain these findings, as the researchers adjusted their analysis for biomarker levels. All these findings support the results of the meta-analysis by Nie et al. [54], who concluded that overweight and obese patients have a significantly reduced risk of CAP mortality despite increased risk of infection.

Studies have also evaluated outcomes other than mortality in patients with obesity and CAP, such as the risk of hospitalization [44], the association with severe CAP or admission to the ICU [55], clinical failure [14], and the probability of sepsis [16]. Researchers have found that in hospitalized patients with CAP, obesity was associated with higher serum C-reactive protein levels and a higher frequency of sepsis (72.4% vs. 64.1%) than in non-obese patients [16]. Furthermore, in a prospective study, Vidal et al. [55] found that the days of stay in the ICU increased significantly in obese people (14 vs. 11.8 days). Additionally, in another study, obesity was associated with an increased risk of clinical failure, although this was not associated with increased mortality [14].

In contrast, other studies have found no statistically significant association between mortality and obese patients with CAP. Wang et al. [51] conducted a retrospective study of 1,652,456 patients with CAP from The United States Nationwide Readmissions Database (NRD) between 2013 and 2014. Mortality was adjusted for potential confounders with propensity score matched analysis. The risk of mortality among obese patients compared to normal BMI patients was 0.75 (95% CI 0.60–0.94). However, after adjustment for confounders, there was no significant difference between study groups.

### 4.3. Obesity and Prognosis in Influenza

Several studies have aimed to determine the risk factors associated with severe manifestations of influenza infection (Table 3) [56,57,58,59,60,61,62,63]. However, severe disease has been defined in diverse ways in the studies, including a range of variables including persistent fever, rapid breathing, altered mental status, signs of pneumonia, acute kidney injury, decompensation of previous chronic diseases, prolonged LOS, ICU admission, or mortality. Most studies have found obesity to be a predictor of poor prognosis in patients with influenza infection.

In this regard, studies have evaluated the risk factors for severity in influenza A (H1N1) pdm09 infection. Ren et al. [56] performed a case-control study with 343 severe hospitalized patients and 343 mild controls in China. The multivariable logistic regression analysis showed that overweight or obesity, age over 60 years, longer time from symptom onset to hospital admission, and chronic disorders were related with an increased risk of severe manifestations of influenza A (H1N1) pdm09. Similarly, Viasus et al. [59] conducted an observational study in hospitalized adults with confirmed influenza A (H1N1) pdm09 virus infection to identify factors associated with severity in 13 hospitals in Spain, defining severity as the composite outcome of ICU admission or hospital mortality. Underlying disease, morbid obesity, younger age, and bacterial coinfection were independent risk factors for severe disease. Another study performed in Sao Paulo, Brazil assessed the risk factors for death in hospitalized patients [57], and found obesity to be among the risk factors associated with poor prognosis from influenza A (H1N1) pdm09. Similarly, Martin et al. [60] conducted a retrospective cohort study of patients aged over 18 years who were admitted to the emergency room or hospitalized with laboratory-confirmed influenza A (H1N1) pdm09. They found that people with obesity more often required hospitalization (OR: 2.93, 95% CI: 1.50–5.71), and more frequently had a prolonged hospital stay (>7 days) compared to non-obese patients. In a systematic review and meta-analysis evaluating the association between obesity and the risk of ICU admission and death among patients hospitalized for influenza A (H1N1) pdm09, obesity doubled the risk of ICU admission and/or mortality [67]. This association was stronger in patients with severe obesity (BMI ≥ 40 kg/m^2^).

Other studies have assessed different seasonal periods to determine predictors of severity in influenza infection [61,62,63]. These studies have evaluated patients infected with different influenza viruses, in addition to the pandemic virus. In a study performed over four seasons (2010/2011, 2011/2012, 2012/2013, and 2013/2014), 777 confirmed cases of influenza A (H1N1) pdm09, influenza A (H3), influenza A, or influenza B were included [61]. Obesity was independently associated with ICU admission but not with hospitalization. Moreover, in a cohort of 66,820 participants aged 65 or older followed up from 1998 to 2012, Zhou et al. [62] also found that obesity aggravates the effect of seasonal influenza on respiratory mortality. Similarly, a study performed during influenza seasons 2010/2011 through 2018/2019 in Spain documented that morbid obesity was also identified as a risk factor for ICU admission, assisted ventilation, and/or death [63].

In contrast, other studies have not found a relationship between obesity and worse prognosis in patients with influenza. Halvorson et al. [64] enrolled 3560 patients (749 children and 2811 adults) to determine the association between obesity and severity of acute respiratory infection (hospital admission, LOS, supplemental oxygen, or antibiotic prescription). This study was conducted over four consecutive winter respiratory seasons (2010–2014). The risk of hospitalization was reduced for overweight and obese adults. However, class 3 obesity (BMI ≥ 40 kg/m^2^) was associated with a higher frequency of supplemental oxygen requirement in adults. Similarly, in another study conducted in the 2017–2018 season, hospitalized adult patients with influenza A and B were divided into those with obesity and controls [65]. The primary composite outcome comprised mortality, vasopressor use, mechanical ventilation, ICU admission, and major complications. In the analyses, obesity was not associated with influenza-related morbidity and mortality despite adjustment for confounders. Related results were found in a study that evaluated hospitalized adults with laboratory-confirmed influenza during the 2012–2013 influenza season in the US [66]. No association was documented between obesity (BMI 30 kg/m^2^ to 34.99 kg/m^2^) or severe obesity (BMI ≥ 35 kg/m^2^) and mortality or ICU admission, even after controlling for comorbidities, demographics, and lifestyle. However, low weight was associated with the development of pneumonia in these patients.

Interestingly, some investigators have evaluated the effect of obesity in both patients with influenza and patients with bacterial pneumonia. Wei et al. [17] evaluated 132,965 patients with influenza and 34,177 patients with pneumonia during the period 2013–2014. The authors found different associations between obesity and mortality in patients with influenza compared with patients with pneumonia: higher BMIs were associated with increased mortality from influenza but with significantly lower mortality from pneumonia (in particular, a BMI from 30 kg/m^2^ to 40 kg/m^2^).

## 5. Conclusions

Acute respiratory infections, including CAP, are common and cause high morbidity and mortality worldwide. Determining the risk factors associated with the development of complications in CAP is important in order to establish the most appropriate site-of-care for patients and the interventions required to improve the prognosis. Few studies have evaluated the impact of undernutrition on the risk and clinical outcomes in adult patients with CAP; however, their results are consistent and show that patients with undernutrition have a higher risk for developing CAP and a decreased survival during the CAP episode. Undernutrition has been frequently documented in patients with CAP and has been associated with high short- and long-term mortality, especially in the elderly. However, a major limitation of the available studies is that the methodologies used to assess undernutrition in CAP patients have varied widely.

Conversely, obesity has been recognized as a factor associated with a lower risk of mortality in CAP. Nevertheless, some of the results of the studies have been inconsistent, especially in relation to the etiology of CAP (bacterial versus viral). Influenza infection, namely that caused by influenza A (H1N1) pdm09, has been associated with a worse prognosis in obese patients. The conflicting findings in the outcomes of influenza infection in obese patients could be due to differences in the influenza strains involved. Other researchers have questioned the use of BMI for assessing obesity, since it is unable to quantify the percentage of body fat and the distribution of adiposity, nor the degree of metabolic alterations that may underlie it [22].

Importantly, studies have documented that screening of patients for risk of malnutrition at hospital admission, followed by nutritional assessment and individualized nutritional interventions, should become part of routine clinical care in hospitals worldwide [19]. However, current data underscore the need to standardize the definition of undernutrition and to consider other aspects when evaluating obesity (for example, muscle mass and central obesity) in CAP studies. In this regard, an international expert group has proposed a framework for the diagnosis of malnutrition that can be used in future studies [21]. In addition, larger studies are also needed to examine the physiological mechanisms that explain the differential impact of malnutrition on outcomes according to different CAP etiologies. Achieving a better understanding of these issues may help in the design of new interventions for improving prognosis of CAP patients with malnutrition.

## Figures and Tables

**Table 1:** Summary of studies on prognosis of CAP and undernutrition.

First Author, Year	Country	Study Design	Number of Patients	Undernutrition Definition	Outcomes
Riquelme et al., 2008 [12]	Chile	Prospective cohort	200	Anthropometric alterations (mid-arm perimeter and TSF) or hypoalbuminemia	Malnutrition was related with LOS (*p* = 0.024) and higher mortality (OR: 2.7, *p* = 0.02). Hypoalbuminemia and low muscle perimeter were associated with higher mortality (OR: 2.7, *p* = 0.015 and OR 4.0, *p* = 0.002, respectively).
Trelles et al., 2020 [13]	United States	Retrospective cohort	89,650	Not defined	PEM was associated with increased mortality (OR: 2.42, *p* < 0.001), septic shock (OR: 3.34, *p* < 0.001), pulmonary embolism (OR: 2.24, *p* = 0.017), mechanical ventilation (OR: 3.13, *p* < 0.001), and prolonged LOS (OR: 3.63, *p* ≤ 0.001).
Matsuo et al., 2020 [38]	Japan	Retrospective cohort	92	Low serum albumin and low BMI	Low BMI was a risk factor for prolonged LOS (OR: 1.18, *p* = 0.042). Low serum albumin was a risk factor for mortality (OR: 81.01, *p* = 0.025).
Hedlund et al., 1995 [39]	Sweden	Prospective cohort	97	PINI, BMI, and TSF	High TSF and low BMI were risk factors for prolonged LOS (*p* < 0.001 and *p* < 0.05 respectively). Low BMI was a risk factor for mortality (*p* < 0.05). PINI and serum albumin were correlated with LOS (*p* < 0.001 and *p* < 0.05 respectively).
Espinoza et al., 2018 [40]	Brazil	Retrospective cohort	802	Not defined	Patients admitted to ICUs with CAP and malnutrition had higher mortality (OR: 2.28, *p* = 0.011).
Shimizu et al., 2020 [41]	Japan	Retrospective cohort	26,098	Second step of the GLIM and BMI	Severe malnutrition in patients ≥ 70 years was associated with mortality (HR: 1.19, *p* ≤ 0.001) and prolonged LOS and 30-day readmission. Patients < 70 years with severe malnutrition had prolonged LOS (HR: 3.27, *p* ≤ 0.001).
Yeo et al., 2018 [42]	Korea	Retrospective cohort	198	Insufficient energy intake, weight loss, loss of muscle mass, loss of subcutaneous fat, localized or generalized fluid accumulation, and diminished functional status measured using handgrip strength.	Malnutrition was associated with 1-year mortality (OR: 3.01; *p* = 0.005) and 2-year mortality (OR: 2.52, *p* = 0.002). Patients admitted to ICUs with CAP and undernutrition had more use of vasopressor and mechanical ventilation rates (11.4% vs. 1.6%, *p* = 0.027 and 12.9% vs. 1.6%, *p* = 0.016, respectively).
Lacroix et al., 1989 [43]	United States	Prospective cohort	5677	Low serum albumin, BMI, arm muscle area, and hemoglobin	Low BMI and arm muscle area were associated with mortality in men (RR: 2.6, *p* = 0.01). Hypoalbuminemia was associated with mortality in women (RR: 3.6, *p* = 0.05)

BMI—body mass index; CAP—community-acquired pneumonia; GLIM—global leadership initiative on malnutrition; HR—hazard ratio; ICU—intensive care unit; LOS—length of hospital stay; OR—odds ratio; PEM—protein energy malnutrition; PINI—prognostic inflammatory and nutritional index; RR—relative risk; TSF—triceps skinfold.

**Table 2:** Summary of studies on prognosis of CAP and obesity.

First Author, Year	Country	Study Design	Number of Patients	Nutritional Status Assessment	Outcomes
Kim et al., 2020 [14]	US	Cohort study	7449	WHO BMI classifications	Higher BMI was associated with higher odds of clinical failure (*p* < 0.001). BMI was significantly protective for mortality between 26.5 and 38.6 kg/m^2^.
Corrales-Medina et al., 2010 [15]	US	Retrospective cohort	266	WHO BMI classifications	Univariate analysis showed a negative association between higher BMI and mortality at 30 days (OR: 0.91, *p* = 0.01).
Singanayagam et al., 2012 [16]	United Kingdom	Prospective cohort	1079	WHO BMI classifications	Obesity was independently associated with reduced 30-day mortality (HR: 0.53). Obese patients had higher median C-reactive protein levels and a higher frequency of sepsis.
Campitelli et al., 2014 [32]	Canada	Retrospective cohort	104,665	WHO BMI classifications	Obesity was a more significant risk factor for acute respiratory infections managed in emergency departments.
Baik et al., 2000 [29]	US	Prospective cohort	104,491	Not defined	Men who gained more than 20 kg of weight were at twice the risk of developing CAP. In women, a direct association was found between BMI > 25 kg/m^2^ and the risk of developing CAP.
Schnoor et al., 2007 [30]	Germany	Case-control	3402	Not defined	Overweight persons have a reduced risk of CAP (OR: 0.6, *p* ≤ 0.001).
Borisov et al., 2022 [44]	US	Secondary analysis of clinical trial	773	WHO BMI classifications	BMI range from 29 to 32 kg/m^2^ was associated with the shortest duration of hospitalization. There was no difference in mortality or ICU admission between BMI groups (*p* > 0.05).
Bertsias et al., 2014 [45]	Greece	Cross-sectional	124	Obese (BMI > 30 kg/m^2^)	No significant variation in the duration of hospitalization between BMI groups. Obesity independently increased the odds for hospitalization due to CAP (OR: 3.4, *p* = 0.037).
Mahendra et al., 2018 [46]	India	Prospective cohort	100	Obese (BMI > 30 kg/m^2^)	From multivariate analysis, investigators found that obesity was independently associated with risk for severe pneumonia (OR: 12.74, *p* < 0.001).
De Miguel-Diez et al., 2022 [47]	Spain	Retrospective cohort	519,750	Not defined	From multivariable logistic regression analysis, the probability of dying in hospital was significantly lower for those with obesity and morbid obesity.
Kahlon et al., 2013 [48]	Canada	Prospective cohort	907	WHO BMI classifications	Obese patients had significantly lower in-hospital mortality after multivariable logistic regression analysis (OR: 0.46, *p* = 0.04).
Braun et al., 2016 [49]	Switzerland	Secondary analysis of multicenter trial	763	WHO BMI classifications	All-cause 6-year mortality was significantly lower in obese patients (HR: 0.641).
Chen et al., 2019 [50]	China	Retrospective	909	WHO BMI classifications	Logistic regression analysis showed that obesity was a risk factor for mortality in patients with CAP (OR: 1.55, *p* = 0.031).
Wang et al., 2019 [51]	US	Retrospective cohort	1,652,456	WHO BMI classifications	No significant difference in mortality of obese patients.
Wang et al., 2022 [52]	China	Retrospective cohort	2327	Overweight/obesity (BMI ≥ 24 kg/m^2^)	Mortality was lowest in the overweight/obesity group and highest in the underweight group (*p* < 0.001). All-cause mortality of overweight/obesity patients was lower (OR: 0.535, *p* = 0.009).
Bramley et al., 2017 [53]	US	Retrospective	2291	WHO BMI classifications	BMI was not associated with ICU admission, but mechanical ventilation was lower among patients who were overweight (OR: 0.51, *p* = 0.02).

BMI—body mass index, CAP—community-acquired pneumonia; HR—hazard ratio; ICU—intensive care unit; LOS—length of hospital stay; OR—odds ratio; US—United States of America; WHO—World Health Organization.

**Table 3:** Summary of studies on prognosis of influenza and obesity.

First Author, Year	Country	Study Design	Number of Patients	Outcomes
Ren et al., 2013 [56]	China	Case-control	686	Overweight (OR: 3.70, 95% CI: 2.04–6.72) and obesity (OR: 35.61, 95% CI: 7.96–159.21) in subjects with influenza A (H1N1) pdm09 were related with severe manifestations.
Ribeiro et al., 2015 [57]	Brazil	Case-control	579	Obesity (OR: 3.06, 95% CI: 1.34–7.00) was a risk factor for death in adults with influenza A(H1N1) pdm09.
Bagshaw et al., 2013 [58]	Canada	Prospective cohort	562	Independent predictors of AKI included obesity (OR: 2.94, *p* < 0.001) in patients with influenza A(H1N1) pdm09.
Viasus et al., 2011 [59]	Spain	Prospective cohort	585	Morbid obesity (OR: 6.7, *p* = 0.001) was an independent factor for severe disease (ICU and/or death) in patients with influenza A(H1N1) pdm09.
Martin et al., 2013 [60]	US	Retrospective cohort	161	Individuals with obesity were more likely to have lower pulmonary disease manifestations (OR: 1.97, 95% CI: 1.05–3.69), be admitted to an inpatient ward (OR: 2.93, 95% CI: 1.50–5.71), and have a lengthy hospital stay (OR: 3.86, 95% CI: 1.03–14.42).
Dimitrijević et al., 2017 [61]	Serbia	Retrospective cohort	777	Obesity significantly increased the risk of ICU admission (OR: 9.80, 95% CI: 3.01–31.93).
Zhou et al., 2015 [62]	Hong Kong	Population-based cohort	66,820	Obesity was an independent risk factor that aggravates the impact of seasonal influenza on respiratory mortality (HR: 1.19, *p* = 0.04).
Derqui et al., 2022 [63]	Spain	Prospective surveillance	3180	Morbidly obese patients showed higher risk of severe outcomes in the 50–64 age group (OR: 3.5, 95% CI: 1.2–10.0). In patients ≥ 80 years, being overweight was associated with decreased risk of severe influenza (OR: 0.6, 95% CI: 0.4–0.9).
Halvorson et al., 2018 [64]	US	Prospective cohort	3560	Risk of hospitalization was decreased with overweight (OR: 0.8, 95% CI: 0.6–1.0), class 1 obesity (OR: 0.7, 95% CI: 0.5–1.0), and class 2 obesity (OR: 0.6, 95% CI: 0.4–0.8). Class 3 obesity was associated with supplemental oxygen requirement (OR: 1.6, 95% CI: 1.1–2.5).
Atamna et al., 2021 [65]	Israel	Retrospective cohort	512	Obesity was not a risk factor for adverse events (OR: 1.3, *p* = 0.5).
Braun et al., 2015 [66]	US	Population-based cohort	9048	No association between obesity or severe obesity and artificial ventilation or ICU admission was found.

HR—hazard ratio; ICU—intensive care unit; OR—odds ratio; US—United States of America.

## Data Availability

Available upon request.
